# Factors Related to Care Competence, Workplace Stress, and Intention to Stay among Novice Nurses during the Coronavirus Disease (COVID-19) Pandemic

**DOI:** 10.3390/ijerph18042122

**Published:** 2021-02-22

**Authors:** Hsiao-Mei Chen, Chien-Chi Liu, Shang-Yu Yang, Yu-Rung Wang, Pei-Lun Hsieh

**Affiliations:** 1 Department of Nursing, School of Nursing Chung Shan Medical University, Taichung City 40201, Taiwan; fionajunlom@gmail.com; 2 Department of Nursing, Chung Shan Medical University Hospital, Taichung City 40201, Taiwan; 3 Department of Nursing, College of Health, National Taichung University of Science and Technology, Taichung City 40343, Taiwan; vickyliu@gm.nutc.edu.tw; 4 Department of Healthcare Administration, College of Medical and Health Science, Asia University, Taichung City 41354, Taiwan; henry879019@yahoo.com.tw; 5 Department of Nursing, Chang Gung University of Science and Technology, Chiayi County 613016, Taiwan; daisy025@gmail.com

**Keywords:** care competence, intention to stay, work stress

## Abstract

The outbreak of coronavirus (COVID-19), a public health emergency of international concern, has made healthcare staff preparation and the nurturing of high-quality and adequate nursing professionals critical issues. This study aimed to explore registered nurses’ competence in nursing care and their intention to stay in their current workplace. In this study, participants who had graduated from different nursing education systems were recruited. The results indicated that nurses’ level of commitment to the workplace and clinical stress were positively correlated with the experience of working with patients. Stepwise regression analysis revealed the following significant predictors for intention to stay: clinical stress, frequency of caring for people with infections, and taking a course on infectious nursing. The novice nurses’ competencies in the areas of pandemic disease care and care for infectious adults depended on the experience of nursing care and nursing competence in their professional careers, which may have impact on the nurses’ intention to stay. Therefore, clinical stress, frequency of caring for patients, and taking nursing courses were correlated with novice nurses’ intention to stay in their professional careers.

## 1. Background

Nurse turnover, work stress, attrition, burnout, and anxiety, among others, have become issues of concern in the healthcare workforce during the coronavirus (COVID-19) pandemic [1,2]. High nurse turnover is costly in terms of temporary replacement, recruitment, loss of productivity, and lower quality of care [3]. However, the COVID-19 pandemic has exacerbated the existing and widespread workforce stress in professional healthcare careers [2]. Numerous individual, organizational, and social factors that affect novice nurses’ intention to stay in their current workplace are associated with patient satisfaction and good quality of care [4,5]. Empirical studies have revealed that nurses’ intention to stay or leave is related to several factors, including personal characteristics, education background, professional development, shift work, work/life/family commitments, organizational vision, work atmosphere, work stress, career opportunities, and quality of work environment [6,7,8]. The cultivation of nursing professionals is paramount for the operation of the clinical medical system and for satisfying the healthcare needs of individuals.

According to the 2016 Asia Workforce Profile revealed by the International Council of Nurses (ICN), the turnover rate of Taiwan’s nursing staff within one year reached 10.5%, ranking third among Asian countries, only after Korea’s 12.6% and Japan’s 10.5% [9]. The Taiwan Union of Nurses Association surveyed the current workforce situation in hospitals in 2016 and found that the average turnover rate of new nursing graduates in 2016 was as high as 18.8%, and that 55.8% of hospitals faced difficulties in recruiting new nursing staff [10]. In recent years, various acute medical and long-term care institutions have experienced nursing staff shortages and have even closed wards or reduced bed numbers as a result. Therefore, reducing the turnover rate of new nursing staff is essential. These high nurse turnover rates are mainly caused by work overload and job stress [5,11,12]. Although the number of new nursing staff should be sufficient every year, poor work environments can result in high turnover rates, leading to instability among new nursing staff who are retained in professional nursing careers.

Given the aforementioned issues, this study aimed to respond to related needs for the promotion of policies and reform of the nursing workplace and to investigate the employment of new nursing staff in Taiwan. We also aimed to examine the changes of nursing competencies in nursing care, the current situation of clinical stress, and the intention to stay in the current workplace under the influence of COVID-19 among new nurses. The results could be provided as a reference for the development of care competence in different nursing academic systems and the cultivation of new nursing staff in professional nursing careers.

## 2. Methods

### 2.1. Research Design

This was a cross-sectional study utilizing a nationwide survey. All new nurses received an electronic questionnaire survey after graduating from different academic nursing programs. Data collection was through this online survey.

### 2.2. Research Sampling

According to the Ministry of Education’s Department of Statistics, there were 14,389 nursing graduates from different nursing academic systems (including 14 general universities, 13 technological universities, and 21 five-year junior colleges) through baccalaureate programs (general and technological universities) and five-year colleges. Probability proportionate to size sampling was conducted based on the proportion of graduates from each school. Of the sample, 46% of the participating nurses were from five-year junior colleges and 54% were in baccalaureate programs (including 44% technological universities and 10% general universities). Four hundred novice nurses were invited to participate in the study after graduation. To be eligible for inclusion, the participants had to be graduates from a department of nursing between the 2017 and 2019 school years, participants in the comparison of nursing competence among nursing students, graduates of that current year in a previous national survey, and needed to have agreed to continue participating in surveys on their nursing care competence.

G*power 3.0 analysis software was utilized to estimate the minimum number of samples required to obtain statistically significant results and the most reasonable sample size to enable a reliable interpretation of the study results and prevent excessive data collection. Based on the use of constants that were suitable for multiple regression analysis (an effect size of 0.15, a significance level of 0.05, and a power of 0.95), a minimum sample of 155 participants was required. In consideration of a 20% data wastage rate, data from 244 novice nurses were collected. To be eligible for inclusion, the participants had to be graduates from a department of nursing between the 2017 and 2019 school years.

### 2.3. Data Collection

Between March and May of 2020, 400 web questionnaires were distributed and 333 valid questionnaires were returned from novice nurses (response rate = 83.25%).

### 2.4. Research Instruments

The variables included in the analysis were demographic data, nursing competence, and intention to stay in the current workplace, and the structured questionnaire was developed in accordance with the study purpose and research framework. Demographic data on the participants’ gender, age, and amount of nursing experience were collected. The current status of nursing competence referred to the related courses and practicum experiences the novice nurses had collected thus far to equip themselves with what was needed for nursing care. The current status of nursing competence was assessed using an author-developed questionnaire to assess factors related to nursing competence based on the essentials of baccalaureate education for professional nursing practice (American Association of Colleges of Nursing (AACN) [13], including ethics, end-of-life care, assessment, risk prevention and quality of care, utilization of resources/programs, resource linkage, acute/chronic/disability care, referral nursing theories, planning, syndrome care, communication, leadership, implementation and utilization of care model. The contents of the self-developed questionnaire were created by referring to the previous literature, and its validity was tested by an expert review for consistency and content validity index (CVI) values for items. The care practicum experience scale was used to explore the novice nurses’ care-related practicum experiences when working in the workplace. There were six items in the scale and a four-point Likert scale was used for scoring, with answers ranging from disagree (1 points) to strongly agree (4 points). The clinical stress scale was developed by [12,14] and includes skill performance, responding appropriately to emergencies, admitting new patients, communication with physicians, developing relationships with colleagues, patients, and families, managing workload demands, organizing and prioritizing duties, doing shift reports, and adjusting to different shifts in the nursing professional careers. The scale analyses novice nurses’ current workforce stress. A total of 15 items were used to test the participants’ level of stress and were scored according to a four-point self-developed Likert scale (in which 1 = strongly disagree, 2 = disagree, 3 = agree, and 4 = strongly agree). A higher score indicated higher self-perceived stress in nursing work. The stay in the nursing workplace scale was used to assess the novice nurses’ willingness to stay in their current position and was scored using a four-point Likert scale (in which 1 = strongly disagree, 2 = disagree, 3 = agree, and 4 = strongly agree). A high average score indicated a low willingness to stay in the job. The expert panel reported a mean individual content validity index of 0.80–0.90, which exceeded the recommended value of 0.78. The scales’ consistency and reliability were found to be high, with a Cronbach’s alpha of 0.95.

### 2.5. Data Analysis

The data were entered into SPSS version 20 (SPSS, Chicago, IL, USA), and demographic characteristics were summarized using descriptive statistics in terms of the frequency distribution. Pearson’s correlation, a t test, multiple regression analysis and one-way analysis of variance with post hoc analysis were employed to detect any association between the variables. Fisher’s least significant difference test was chosen as the most appropriate post hoc method to detect differences for all pairwise comparisons in this data set. The statistical significance was set at *p* ≤ 0.05.

### 2.6. Ethical Considerations

Ethical approval for the study was obtained from the Human Research Ethics Council (HREC-E-105-306). To ensure that research ethics were adhered to, the researcher explained the research purpose and process to the participants and informed them about the protection of their personal rights. Informed consent forms were provided before data collection.

## 3. Results

### 3.1. Personal and Demographic Characteristics of the Participants

This study analyzed the data of 333 participants (92.8% women) who completed the questionnaire. Among these participants, 44.58% worked in the medical center, 16.87% worked in the regional hospital, and 8.43% worked in the district hospital. In terms of the participants’ education level, those graduated from general universities accounted for 16.9%, technological universities accounted for 66.2%, and five-year junior colleges accounted for 16.9%. Among such institutions, technological universities accounted for the majority. The results revealed that the frequency of providing care to patients with infections helped most participants improve their competence in communicating with such patients (55.4% chose agree and 39.8% chose strongly agree). Furthermore, 94% agreed or strongly agreed that work experience helped improve their ability to provide care to patients with infections. Moreover, 56.6% agreed (and 36.1% strongly agreed) that the experience would help them apply professional knowledge to the provision of care to patients with infections in the future. However, 66.3% of the participants were not particularly interested in providing care to infectious people during their nursing career (25.3% were interested). During their nursing work, 87.9% of the participants were interested in providing care to patients. The personal attributes of the subjects are shown in Table 1.

### 3.2. Nursing Competence

The average score of the overall nursing competencies for the nurses was between agreement and strong agreement (mean = 3.11, SD = 0.52). The highest average score (mean = 3.30, SD = 0.51) was for “Ethics”, and the lowest average score was for “Utilization of Care Model” (mean = 2.93, SD = 0.66). The results are presented in Table 2.

### 3.3. Clinical Stress

The mean for workforce stress of the participants was ranked between disagreement and agreement (mean = 2.31, SD = 0.48). The highest score was obtained in “Performing CPR on patients is stressful” (2.69 ± 0.92), followed by “Excessive workload during a fixed period is stressful” (mean = 2.57, SD = 0.80) and “The sudden change in a patient’s condition makes me feel stressed” (mean = 2.52, SD = 0.82). Please see Table 3 for details.

### 3.4. Intention to Stay in Their Current Position

The average score of the willingness for the participants to stay in their job was 2.00 ± 0.46, which was close to disagreement. The item “I am willing to continue my current nursing work for the next year” scored the highest (mean = 2.87, SD = 0.94), followed by the items “I may change my job if there is an opportunity for another job that suits me” (mean = 2.53, SD = 0.92) and “I am actively looking for a nursing job at another medical institution” (mean = 2.26, SD = 0.86). The item “I am unsatisfied with my current wage and am therefore unwilling to continue this job” (mean = 1.81, SD = 0.91) scored the lowest, followed by “I am not satisfied with the current content of my job and am therefore unwilling to continue this job” (mean = 1.84, SD = 0.80) and “I am not satisfied with the system in my current work unit and am therefore unwilling to continue this job” (mean = 1.86, SD = 0.85). Details are presented in Table 4.

### 3.5. Correlations between Various Attributes and Scale Items

The study analyzed the correlations between demographic attributes, service institution attributes, current status of care for patients, ability to provide care to patients, willingness to stay in their current position, and clinical stress. The results revealed that education level was positively correlated with the ability to provide care to patients (F = 7.44, *p* < 0.001) and willingness to stay in their current position (F = 6.16, *p* < 0.001). Frequency of providing care to people with suspected COVID-19 infections (e.g., nurses had to assist with the continuous viral nucleic acid testing specifications, isolation and management measures for patients with suspected symptoms) in the workplace was positively correlated with willingness to stay in their current position (r = 0.25, *p* < 0.001) and clinical stress (r = 0.23, *p* < 0.001). For participants, taking a course related to providing pandemic care after starting work was positively correlated with willingness to stay in their current position (r = 0.14, *p* = 0.01). Interest in providing care to patients during nursing work was positively correlated with the ability to provide care to patients (r = 0.20, *p* < 0.001) and negatively correlated with clinical stress (r = −0.17, *p* = 0.002). Furthermore, for the participants, clinical stress was positively correlated with willingness to stay in their current position (r = 0.48, *p* < 0.001) during the COVID-19 pandemic.

### 3.6. Factors Influencing Novice Nurses’ Intention to Stay in Their Current Position

According to the analysis results of the factors affecting nurses’ willingness to stay in their current position, we included significant variables for various academic systems. The results (see Table 5) revealed that the following variables were significant: frequency of providing care to patients in the workplace, having taken a course on providing care related to pandemic prevention after starting work, willingness to provide services, and clinical stress. The stepwise analysis of the factors influencing clinical stress (B = 0.41, *β* = 0.44, *p* = 0.000) revealed that having taken a course related to providing care to patients after starting work (B = 0.12, *β* = 0.04, *p* = 0.005) and frequency of providing care to infection patients in the workplace (B =0.12, *β* = 0.03, *p* = 0.000) were the primary predictors and could explain 23% of the variance in the novice nurses’ intention to stay in their current professional nursing careers.

## 4. Discussion

This study explored novice nurses’ competence in clinical care and examined its relationship with their clinical stress and intention to stay in the current workplace. This study demonstrated the factors that were associated with clinical stress, frequency of providing care to adults/people with diseases in the workplace, and having taken the course related to providing care to patients after starting work.

The demographic characteristic analysis revealed that the majority of the participants in the study were women who were currently working in medical center settings, and the majority of these nurse participants were relatively young (an average age of 22.33 years) compared to the national workforce [9]. A comparison of studies on the nursing workforce in international acute illness units and medical centers indicated that younger nurses typically work in acute care units [12]. The results of the present study revealed that the majority of the novice nurses’ workplaces varied significantly among the transition shock [2,11,12]. Nurses’ reasons for choosing their workplace is a relatively new research topic and most studies have focused on job satisfaction during their professional nursing careers [7,15].

The novice nurses had the competence to provide quality care related to patient ethics, end-of-life care, and risk prevention. However, the novice nurses had the lowest average score in “utilization of care models, implementation, and leadership in the team,” indicating a lack of adequate practicum experience in leading care teams. The results indicated that the novice nurses who took infection care related courses in baccalaureate nursing programs were more familiar with relevant concepts and had greater awareness of care related concepts.

The present study also revealed that the participants’ education level, intention to stay in their current position, and clinical stress were positively correlated with competence in care, and that clinical stress was positively correlated with the willingness to stay in their current position. Studies have indicated that taking courses related to pandemic prevention during the study period may be correlated with willingness to serve, and that those with a good education experience, such as taking courses, would have greater willingness to care for patients [12,16]. The results indicated that the participants were willing to stay in their current nursing workplace and that care competence was positively correlated with intention to stay. Intention to stay was negatively correlated with clinical stress. The stepwise regression analysis revealed the following significant predictors of intention to stay: clinical stress, frequency of caring for infectious patients in the workplace, receiving nursing courses in the work field, and the frequency of care for patients in professional nursing careers.

When faced with emotional distress and other problems in the workplace, novice nurses can adjust themselves and use a positive attitude, which greatly increases their intention to stay [17,18]. Our study revealed that novice nurses with higher resilience had a higher intention to stay during the coronavirus (COVID-19) pandemic. This result implied that nursing work is highly stressful and has many uncertainties that can generate negative emotions and affect novice nurses’ physical and mental health. In addition, high levels of workplace stress reduce job satisfaction, increasing their turnover intention [2,12,19,20,21,22]. Hospitals are regarded as a high-risk workplace with high professional complexity and nurses can be easily affected by work frustration in hospital environments. Nurses who are less experienced or are not equipped with high levels of resilience and professional skills will have a reduced intention to stay in a hospital environment.

Given global nursing staff shortages, it is necessary to intervene appropriately to alleviate work stress and frustration, thereby improving the nursing workflow and helping solve the problems of staff shortages [2,7,21,23,24]. Therefore, nursing supervisors must provide positive support to their nursing staff, strengthen their nurses’ competence to help them overcome highly stressful challenges in clinical contexts, seek to lower the factors causing work frustration, establish sound professional training programs and support channels, and improve the professional competency of nursing staff.

### 4.1. Limitations

The national survey results depended on the status of the respondents when they filled out the questionnaire; thus, the results cannot entirely reflect their real intention to stay in their current workplace. The respondents filled in their statements subjectively, making it difficult to determine the actual intention of the participants. Moreover, this was a quantitative study, and it was not easy to understand the novice nurses’ perception of their intention to stay in their professional nursing careers. Qualitative interviews should be conducted to elucidate the influencing factors revealed in this study.

### 4.2. Relevance to Clinical Practice

Clinical stress, frequency of caring for patients with infectious diseases (such as the newly discovered coronavirus), and receiving a course on nursing were correlated with novice nurses’ intention to stay in their current workplace. Their willingness to provide care services to patients should be increased because of population aging and the increasing demands caused by pandemic issues. Novice nurses should receive professional training related to older adults or people with pandemic diseases, which is crucial to the continued improvement of nursing care quality.

## 5. Conclusions

Based on the results of this study, the following suggestions regarding nursing education, practice, research, and policy are presented. These results could arise from the educational training provided for such novice nurses in their research hospitals and the abilities of adjustment to pressure and care-related communication the nurses developed in such courses. Novice nurses should be helped to develop stronger coping strategies and enhance their resilience after they encounter difficulties or are emotionally vulnerable in their professional nursing careers.

Nurses should be equipped with the knowledge and skills related to clinical care, and pandemic disease and related courses should be integrated with clinical and practical contexts in which nursing practical training is conducted to increase novice nurses’ frequency of contact with older adults and people with pandemic infections, thereby helping them with their future professional nursing careers.

Considering population aging, patient demands for care, and the pivotal role that nurses play in clinical care, policymakers should include courses on healthcare in the nursing curriculum as crucial professional training. It is suggested that the Ministry of Health and Welfare continue to cultivate excellent nursing professionals in sufficient numbers.

## 6. Implications for Nursing Management

Novice nurses should provide care to older adults and people with infections more often so as to accumulate experience in a clinical context. Their willingness to provide long-term care services to patients should also be increased to address the needs of an aging population and the increasing demands of long-term care and COVID-19 pandemic issues. To cope with the trend, new nurses with professional training in clinical care are required to improve care quality in clinical settings.

The results of this study showed that pressure is common for novice nurses who are working in clinical settings. Maintaining the care competence of novice nursing staff is essential to providing services to patients and an aging population. Nurse leaders should pay attention to the work stress and the related factors of novice nurses. Furthermore, nurse leaders should guide nurses to adjust their psychological state scientifically. Nursing managers should try their best to provide safe working conditions for novice nurses and to raise the enthusiasm and meticulousness of nurses. Leisure activities and training on how to relax should be properly arranged to help novice nursing staff reduce stress.

## Figures and Tables

**Table 1:** Personal attributes of the subjects (N = 333).

Variables	Categories	N	(%)
Gender			
	Female	309	92.8
	Male	24	7.2
Education			
	Junior college	56	16.9
	General universities	56	16.9
	Technological universities	221	66.2
	Graduate school and above	0	0
Workplace			
	Medical center	150	45.0
	Regional hospital	88	26.4
	District hospital	63	18.9
	Others (e.g., community service center)	32	9.7
Experience in nursing practicum (year)			
	<1	152	45.6
	1–2	98	29.5
	>2	83	24.9

**Table 2:** Difference analysis of the nurses’ competence in nursing care (N = 333).

Core Competence	Mean	SD
Nursing competencies (overall)	3.11	0.55
Ethics	3.30	0.51
End-of-life care	3.20	0.49
Assessment	3.20	0.53
Risk prevention and quality of care	3.18	0.54
Utilization of resources/programs	3.14	0.52
Resource linkage	3.13	0.51
Acute and chronic co-morbid and disability care	3.11	0.54
Referral	3.10	0.57
Nursing theories and concepts	3.10	0.48
Planning	3.10	0.53
Syndromes care	3.06	0.57
Communication	3.06	0.52
Leadership	3.04	0.67
Implementation	3.02	0.58
Utilization of Care Model	2.93	0.66

**Table 3:** Clinical stress scale results (N = 333).

Item	Mean	SD
**Overall**	2.31	0.48
1. Admitting new patients makes me feel stressed.	2.42	0.70
2. Communicating with doctors makes me feel stressed.	2.18	0.63
3. Communicating with colleagues makes me feel stressed.	2.04	0.61
4. Communicating with patients or their family members makes me feel stressed.	2.05	0.56
5. I am afraid to hurt patients because of negligence while providing care (e.g., needle-stick injury, giving the wrong medicine, or not using a bed rail).	2.30	0.78
6. Being unfamiliar with how to use medical instruments on patients makes me feel stressed.	2.19	0.67
7. Insufficient professional knowledge and being unable to identify dangerous signs in a patient make me feel stressed.	2.34	0.63
8. Performing CPR on patients is stressful.	2.69	0.92
9. Writing reports is stressful.	2.35	0.80
10. Night shifts, which place a burden on my body, make me feel stressed.	2.06	0.83
11. The sudden change in a patient’s condition makes me feel stressed.	2.52	0.82
12. Excessive workload during a fixed period is stressful.	2.57	0.80
13. Work allocation problems that increase my care workload make me feel stressed.	2.25	0.81
14. Fatigue and an inability to take time off to rest make me feel stressed.	2.45	0.87
15. Shift work makes me feel stressed.	2.28	0.83

**Table 4:** Intention to stay in the current position (N = 333).

Item	Mean	SD
**Overall**	2.00	0.46
1. I am considering quitting my current job within the next year.	1.88	0.93
2. I am willing to continue my current nursing work for the next one year.	2.87	0.94
3. I am unwilling to do a nursing job again.	1.63	0.74
4. I will seek another nursing job at another medical institution.	2.26	0.86
5. I would like to leave my current emergency unit and seek a long-term care job opportunity.	1.48	0.63
6. I am considering quitting my current job and would like to find a nursing-related job.	1.88	0.85
7. I am not satisfied with my current wage and am therefore unwilling to continue in the job.	1.81	0.91
8. I am not satisfied with the current content of my job and am therefore unwilling to continue in the job.	1.84	0.80
9. I am not satisfied with the system in my current work unit and am therefore unwilling to continue in the job.	1.86	0.85
10. I may change my job if another job opportunity arises that suits me.	2.53	0.92

**Table 5:** Factors affecting novice nurses’ intention to stay in their current position (N = 333).

				Unstandardized Coefficients		Standardized Coefficients		Collinearity Statistics	
	R	R Square	Adjusted R Square	Β	SEB	β	t	Tolerance	VIF
Model	0.48	0.23	0.23						
(Constant)				1.01	0.11		9.10		
Workplace stress				0.41 **	0.05	0.44	8.59	0.90	1.11
Having taken the course related to pandemic prevention				0.12 *	0.04	0.14	2.80	0.96	1.05
Frequency of providing care to infection patients in the workplace				0.12 **	0.03	0.26	3.69	0.48	2.10

* Significant at *p* < 0.05; ** Significant at *p* < 0.01. Statistics were based on a stepwise regression analysis.

## Data Availability

The data presented in this study are available on request from the corresponding author.
